# Public awareness and satisfaction with the centre antipoison du quebec (CAPQ): a population-based survey

**DOI:** 10.1007/s43678-026-01186-3

**Published:** 2026-06-28

**Authors:** Maude St-Onge, Audrée Elliott, Win Sweeris Vong, Megan Bergeron

**Affiliations:** 1 Centre antipoison du Québec, CIUSSS de la Capitale-Nationale, Québec, QC Canada; 2 Department of Family and Emergency Medicine and Department of Anesthesiology and Critical Care, Faculty of Medicine, Université Laval, Québec, QC Canada; 3 CHU de Québec – Université Laval, Québec, QC Canada; 4 Pavillon Jeffrey-Hale, Québec, QC Canada; 5 Department of Biomedical Sciences, Université Laval, Québec, QC Canada

**Keywords:** Poison control centers, User satisfaction, Quality improvement, Surveys and Questionnaires, Awareness, Centres antipoison, Satisfaction des utilisateurs, Amélioration de la qualité, Enquêtes et questionnaires, Sensibilisation

## Abstract

**Objectives:**

To describe public awareness of the Centre antipoison du Québec (CAPQ), expectations regarding acute poisoning management and service delivery, and satisfaction among prior users.

**Methods:**

We conducted a cross-sectional, bilingual online survey of Quebec residents aged ≥ 14 years between April 13, 2024, and June 27, 2025. The questionnaire collected demographic data, assessed awareness and prior use of the CAPQ, explored past poisoning events and expectations for acute poisoning management, and measured satisfaction among previous users using a 5-point Likert scale, with open-ended questions for qualitative input. Participants were recruited through convenience and snowball sampling via public settings, social media, and CIUSSS newsletters. Descriptive statistics summarized responses and explored trends.

**Results:**

A total of 611 respondents were included; 541 (88.5%) were aware of the CAPQ, including 152 prior users, while 70 (11.5%) were unaware. Unawareness was more common among females (60.0%), non-healthcare workers (91.4%), respondents with a non-North American ethnic background (34.2% vs. 10.1%), and those not primarily French-speaking (12.9% vs. 3.0%). Regional variation was observed, with Capitale-Nationale contributing the largest number of unaware respondents (35/70) and Laurentides showing the highest proportion (26.7%). Among respondents without prior CAPQ use who reported a poisoning event, most contacted emergency services or presented to the emergency department. Expectations were similar among aware and unaware respondents, particularly for acute poisoning management, with nurses expected to respond within five minutes. Among prior users, overall satisfaction was high across all domains, though communication and outreach were identified as areas for improvement. Key service expectations included rapid 24/7 access (76.9%), responder expertise (69.9%), and clear, adapted recommendations (56.1%). Most respondents preferred French (84.9%), telephone communication (94.1%), and follow-up calls (81.7%).

**Conclusions:**

User satisfaction with the CAPQ was high, but gaps in public awareness and service expectations remain. These findings highlight opportunities to improve accessibility, communication and outreach strategies.

**Supplementary Information:**

The online version contains supplementary material available at https://doi.org/10.1007/s43678-026-01186-3.

## Résumé

**Objectifs:**

Décrire la sensibilisation du public au Centre antipoison du Québec (CAPQ), les attentes concernant la gestion des intoxications aiguës et la prestation de services, ainsi que la satisfaction des utilisateurs antérieurs.

**Méthodes:**

Nous avons mené une enquête en ligne transversale et bilingue auprès de résidents du Québec âgés de ≥14 ans entre le 13 avril 2024 et le 27 juin 2025. Le questionnaire a permis de recueillir des données démographiques, d’évaluer la connaissance et l’utilisation antérieure du CAPQ, d’explorer les événements d’intoxication passés et les attentes en matière de gestion des intoxications aiguës, et de mesurer la satisfaction des utilisateurs précédents à l’aide d’une échelle de Likert à 5 points, avec des questions ouvertes pour l’apport qualitatif. Les participants ont été recrutés par commodité et par échantillonnage de boules de neige via des lieux publics, les réseaux sociaux et les bulletins d’information du CIUSSS. Les statistiques descriptives résument les réponses et explorent les tendances.

**Résultats:**

Un total de 611 répondants ont été inclus; 541 (88,5 %) étaient au courant du CAPQ, y compris 152 utilisateurs précédents, tandis que 70 (11,5 %) n’étaient pas au courant. L’ignorance était plus fréquente chez les femmes (60,0 %), les non-travailleurs de la santé (91,4 %), les répondants d’origine ethnique non nord-américaine (34,2 % contre 10,1 %) et ceux qui n’étaient pas principalement francophones (12,9 % contre 3,0 %). Une variation régionale a été observée, la Capitale-Nationale ayant contribué au plus grand nombre de répondants ignorants (35/70) et les Laurentides affichant la proportion la plus élevée (26,7 %). Parmi les répondants n’ayant pas utilisé le CAPQ qui ont signalé un événement d’empoisonnement, la plupart ont contacté les services d’urgence ou se sont présentés au service des urgences. Les attentes étaient similaires chez les répondants conscients et inconscients, en particulier pour la prise en charge des intoxications aiguës, les infirmières étant censées répondre dans les cinq minutes. Parmi les utilisateurs précédents, la satisfaction globale était élevée dans tous les domaines, bien que la communication et la sensibilisation aient été identifiées comme des domaines à améliorer. Les principales attentes en matière de services comprenaient un accès rapide 24/7 (76,9 %), l’expertise des intervenants (69,9 %) et des recommandations claires et adaptées (56,1 %). La plupart des répondants préfèrent le français (84,9 %), les communications téléphoniques (94,1 %) et les appels de suivi (81,7 %).

**Conclusions:**

La satisfaction des utilisateurs à l’égard du CAPQ était élevée, mais il subsiste des lacunes dans la sensibilisation du public et les attentes en matière de services. Ces résultats mettent en évidence les possibilités d’améliorer l’accessibilité, la communication et les stratégies de sensibilisation.

## Clinician’s capsule

**Table:** 

***What is known about the topic?***
A prior study found that the Centre antipoison du Québec fulfilled its perceived missions among 1st line healthcare professionals, whereas public awareness and user expectations remain largely unexplored.
***What did this study ask?***
What are public awareness, expectations and satisfaction regarding services provided by the CAPQ.
***What did this study find?***
Gaps in public awareness and expectations of CAPQ services highlight the need for improved communication and outreach.
***Why does this study matter to clinicians?***
Improve visibility and accessibility to poison centers and support safe management of toxic exposures outside the emergency department.

## Introduction

Poisoning remains a significant public health concern in Canada, with over 79,000 emergency department visits in 2018 from unintentional exposures and annual costs exceeding $2.6 billion [1]. Previous studies have shown that poison centers can substantially reduce emergency visits and associated economic burden through remote guidance [2–4]. Poison control centers play a central role in managing and preventing poisoning events by providing real-time guidance to the public and healthcare professionals. They also contribute to public health surveillance through systematic collection of poisoning data. Many exposures can be managed safely with remote expert advice, underscoring the importance of public awareness and appropriate use of these services [1].

The Centre antipoison du Québec (CAPQ) is the sole provincial poison center in Quebec, offering 24/7 bilingual consultation for toxic exposures. In 2024, the CAPQ managed over 50,000 cases, two-thirds involving the public [5]. A previous evaluation of the CAPQ in 2016 focused mainly on healthcare professionals, reporting overall satisfaction alongside areas for improvement [6]. However, despite the public’s central role in poisoning events, studies examining public awareness, expectations, and experiences with poison center services remain limited.

This study evaluates public awareness of the CAPQ, knowledge of its services, expectations regarding acute poisoning management and service delivery, and satisfaction among prior users. It aims to identify the needs and expectations of Quebec residents, and to assess perceived strengths and areas for improvement.

## Methods

### Study design and setting

We conducted a cross-sectional survey to assess public awareness of, expectations for, and satisfaction with services provided by the CAPQ. The questionnaire was developed by the study team in collaboration with the CAPQ clinical and administrative experts. It included multiple-choice questions, with open-ended questions following satisfaction and expectations items. Multiple answers were also permitted. Satisfaction was measured using a 5-point Likert scale (1 = not at all satisfied; 5 = very satisfied). The survey underwent bidirectional French–English translation and pretesting to ensure clarity and linguistic consistency. Completion time was approximately 15 min, and respondents were routed based on prior CAPQ use and awareness. The online questionnaire was administered via Microsoft Forms from April 13, 2024, to June 27, 2025. The study was approved by the CIUSSS de la Capitale-Nationale Research Ethics Board, and participants provided informed consent. The survey forms and the survey posters can be found in the supplementary material.

### Sample and population

A target sample size of 1,067 was calculated to achieve a 3% margin of error at 95% confidence. Recruitment was halted when response rates plateaued and the sample was deemed sufficient for descriptive objectives. Participants were recruited through convenience sampling with snowball dissemination. QR codes were distributed in drugstores, schools, and youth centers and shared via social media and CIUSSS newsletters. Eligible participants were individuals aged 14 years or older residing in Quebec, regardless of prior CAPQ use. Respondents were excluded if they did not provide consent, were under 14 years of age, or did not reside in Quebec.

### Outcome measures

Collected variables included demographic characteristics (age, gender, region, ethnicity, and employment in healthcare). Awareness and prior use of the CAPQ services were assessed. Based on prior CAPQ use, respondents were divided into two groups: Group A (no prior use) and Group B (previous users). Among Group A, awareness of the center and behaviors during past poisoning experiences were assessed. Expectations regarding acute poisoning management were evaluated, including preferred responder type, acceptable waiting times, and the perceived importance of anonymity. For respondents aware of the CAPQ, knowledge of available services was also assessed. Among Group B, prior call experience was assessed through overall satisfaction, responder professionalism, clarity of recommendations, and the perceived relevance of the advice received. All respondents were then asked about expectations relevant to quality improvement, including preferred service modalities, responder characteristics, communication preferences, follow-up, and outreach channels. The survey flow diagram is provided in the supplementary material S1.

### Data analysis

Questionnaires, with missing information except for the residential region, were included. Results were summarized and reported using descriptive statistics (raw numbers and proportions). Responses to open-ended questions were reviewed and categorized into themes.

## Results

A total of 611 respondents were included. Overall, 541 (88.5%) reported being aware of the Centre antipoison du Québec (CAPQ), including 152 previous users, while 70 (11.5%) were unaware. There were no notable differences in awareness across age groups. Most unaware respondents were female (60.0%) and not employed in the healthcare sector (91.4%). Additionally, 34.2% reported a non-North American ethnic background, compared with 10.1% in the aware group, and a higher proportion did not primarily speak French (12.9% vs. 3.0%) (Table 1).

**Table 1:** Respondent’s demographic characteristics

Previous awareness of the CAPQ	Aware (*n* = 541)	Unaware (*n* = 70)	Total (611)
	88.5%	11.5%	
Prior use of CAPQ services			
	28.1%	0	28.1%
Age group			
14–19	3.5%	20.0%	5.4%
20–29	19.0%	20.0%	19.1%
30–39	15.1%	5.7%	11.8%
40–65	51.2%	11.4%	39.1%
66–75	18.2%	21.4%	15.9%
75 +	8.4%	21.4%	8.7%
Sex assigned at birth			
Male	24.2%	40.0%	26.0%
Female	75.4%	60.0%	73.6%
Gender identity			
Cisgender	95.4%	95.7%	95.4%
Transgender	0.6%	0	0.5%
Non-binary	1.3%	1.4%	1.3%
Ethnic background			
North American	88.9%	62.9%	85.9%
European	5.5%	17.1%	6.9%
African	1.3%	5.7%	1.8%
Asian	1.3%	5.7%	1.8%
Other^a^	2.0%	5.7%	2.5%
Healthcare workers			
Yes	41.0%	8.6%	37.3%
No	58.6%	91.4%	62.4%
Language preference			
French	86.0%	78.6%	85.1%
English	3.0%	12.9%	4.1%
No preference	11.1%	12.9%	11.3%
Area of residence in quebec			
Bas-Saint-Laurent (01)	0.9%	0	0.8%
Gaspésie-Îles-de-la-Madeleine (11)	1.8%	0	1.6%
Saguenay-Lac-Saint-Jean (02)	2.6%	0	2.3%
Nord-du-Québec (10)	0.7%	0	0.7%
Capitale-Nationale (03)	40.7%	50.0%	41.7%
Chaudière-Appalaches (12)	12.6%	12.9%	12.6%
Mauricie (04)	9.8%	1.4%	8.8%
Centre-du-Québec (17)	7.8%	1.4%	7.0%
Estrie (05)	2.0%	1.4%	2.0%
Montréal (06)	7.2%	12.9%	7.9%
Laval (13)	1.7%	4.3%	2.0%
Lanaudière (14)	1.8%	4.3%	2.1%
Laurentides (15)	2.0%	5.7%	2.5%
Montérégie (16)	3.7%	4.3%	3.8%
Outaouais (07)	1.8%	0	1.6%
Abitibi-Témiscamingue (08)	1.1%	0	1.0%
Côte-Nord (09)	1.7%	1.4%	1.6%

^a^Other ethnic backgrounds included Latin, South and Central American and unspecified origins

Given the noticeable variation in sample size across regions, we explored regional awareness using two complementary approaches: the absolute number of unaware respondents and the proportion of unaware respondents within each region. The largest numbers of unaware respondents were from Capitale-Nationale (35/70; 50.0%), Chaudière-Appalaches (9/70; 12.9%), and Montréal (9/70; 12.9%) (Table 1). When examining proportions within each region, the highest rates of unawareness were observed in Lanaudière (3/13; 26.7%), Laval (3/12; 25.0%), and Laurentides (4/15; 23.1%) (Supplementary Table S2).

### Group A: respondents without prior CAPQ use

Among respondents unaware of the CAPQ (*n* = 70), 4 reported a past poisoning experience, 1 called 911 and 2 went to the emergency department. Expectations regarding acute poisoning management revealed that nurses (37) and pharmacists (29) were the most expected responders. A waiting time of 1–5 min was considered reasonable by 37 people, and 51 reported that anonymity was not important (Table 2).

**Table 2:** Responses to questions of respondent unaware of the CAPQ among group A (*N* = 70)

Self or close contact experienced a poisoning incident among unaware respondents		
	Yes	No
Number of participants (*n* = 70)	4	66

Actions taken during poisoning incident (more than one answer can be selected)					
	Nothing	Called 811	Called 911	Called a pharmacist	Went to the emergency department
Number of participants (*n* = 4)	2	1	1	1	2

Preferred channels for outreach							
	Television	Radio	Journal	Facebook	Instagram	TikTok	Other^a^
Number of participants	31	16	15	27	27	16	7

Expected type of professional when calling						
	Doctor	Resident	Nurse	Pharmacist	Receptionist	Other^b^
Number of participants	22	11	37	29	24	5

Perceived reasonable waiting time				
	Less than a minute	1–5 min	5–30 min	More than 30 min
Number of participants	20	37	11	2

Importance of anonymity when calling		
	Yes	No
Number of participants	19	51

^a^Proposed outreach alternatives included digital communication methods, institutional channels, posters in public spaces, and telephone calls

^b^Expected types of professional when calling the CAPQ also included students

In the aware group (*n* = 389), 65.6% knew the center manages all types of poisoning exposures, 36.5% were aware it could notify a nearby hospital, and 52.7% were aware of the CAPQ website. 48 reported a past poisoning experience, 13 called 911, and 19 called the emergency department. In the event of a poisoning, nurses (35.2%) and pharmacists (31.9%) were also most frequently expected to answer calls. A waiting time of 1–5 min was considered reasonable by 63.0%, and 85.9% reported that anonymity was not important (Table 3).

**Table 3:** Responses to questions of respondents aware of the CAPQ among group A (*N* = 389)

Aware that the center responds to all types of poisoning exposure		
	Yes	No
Number of participants	65.6%	34.4%

Aware that the center can notify a nearby hospital for emergency care		
	Yes	No
Number of participants	36.5%	63.5%

Aware of the CAPQ website		
	Yes	No
Number of participants	52.7%	47.3%

Self or close contact experienced a poisoning incident among aware respondents		
	Yes	No
Number of participants (*n* = 389)	12.3%	87.7%

Actions taken during poisoning incident (more than one answer can be selected)					
	Nothing	Called 811	Called 911	Called a pharmacist	Went to the emergency department
Number of participants (*n* = 48)	18.8%	22.9%	27.1%	6.3%	39.6%

Expected type of professional when calling						
	Doctor	Resident	Nurse	Pharmacist	Receptionist	Other^a^
Number of participants	18.8%	4.1%	35.0%	31.6%	1.0%	8.7%

Perceived reasonable waiting time				
	Less than a minute	1–5 min	5–30 min	More than 30 min
Number of participants	27.0%	63.0%	10.0%	0

Importance of anonymity when calling		
	Yes	No
Number of participants	14.1%	85.9%

^a^Expected types of professional when calling the CAPQ also included skilled staff, any healthcare professional, and specialized poison experts

### Group B: respondents with prior CAPQ use (Table 4)

**Table 4:** Satisfaction among participants who previously used the CAPQ’S services (*N* = 152)

	Level of satisfaction				
	1 (Not at all satisfied)	2	3	4	5 (Very satisfied)
Overall satisfaction with the call	2.0%	1.3%	2.6%	9.2%	84.9%
Satisfaction with attentiveness during the call	2.6%	0.7%	2.0%	12.5%	82.2%
Satisfaction with professionalism of the responder	2.6%	1.3%	1.3%	9.2%	85.5%
Clarity of the recommendations received	1.3%	1.3%	2.6%	11.2%	83.6%
Relevance and applicability of the recommendations (*n* = 150)	1.3%	0	4.6%	13.3%	80.7%

A total of 152 respondents reported prior CAPQ use, with healthcare and non-healthcare workers similarly represented (Supplementary Table S3). User satisfaction ratings are presented in Table 4, with corresponding user comments provided in Supplementary Table S4. Most respondents reported being very satisfied overall (84.9%). High satisfaction was also observed for responder attentiveness (82.2%), with comments frequently highlighting attentive, calm, and reassuring staff, and many respondents reporting feeling well supported during stressful situations. Negative feedback mainly concerned perceived lack of empathy or interruptions. Similarly, 85.5% reported being very satisfied with responder professionalism, although a few comments mentioned a condescending tone, feeling judged for minor exposures, and occasional long wait times or voicemail redirection. Satisfaction was also high for clarity of recommendations (83.6%) with most respondents describing the advice as clear, understandable, and reassuring. Negative comments primarily concerned overly detailed explanations, rapid speech, or the need for more concise instructions and clearer follow-up guidance. Finally, 80.7% reported being very satisfied with the relevance of the advice. Comments on the applicability of recommendations were largely positive, with respondents describing the guidance as relevant, reassuring, and easy to apply to their situations.

### All respondents: expectations relevant to quality improvement (Table 5)

**Table 5:** Responses to quality improvement questions of all respondent, aware and unaware of the CAPQ (*N* = 611)

Main expectations for the service				
	Suited communication tools	Poisoning prevention tools	Fast call response at any time	Other
Number of participants	10.3%	11.3%	76.9%	1.5%

Expectations from the call responder				
	Active listening	Clear, simple language	Responder expertise	Other
Number of participants	16.4%	12.1%	69.9%	1.6%

Expectations for recommendations			
	Follow-up call	Fast advice on hospital need	Clear and adapted recommendations
Number of participants	5.9%	38.0%	56.1%

Preferred mean of communication					
	Telephone call	Video call	Text message	E-mail	Other
Number of participants	94.1%	16.7%	24.5%	12.6%	1.0%

Preferred response language			
	French	English	No preference
Number of participants	84.9%	11.6%	6.7%

Interest in receiving a follow-up call after home monitoring advice			
	Yes	No	No preference
Number of participants	81.7%	11.6%	6.7%

Preferred methods and channels for awareness campaign					
	Website resources	Short videos	Interactive games	Social media	Other
Number of participants	27.5%	19.3%	2.0%	48.6%	2.6%

Across all respondents (*n* = 611), the most frequently reported primary expectation was rapid 24/7 call response (76.9%), followed by poisoning prevention tools (11.3%) and adapted communication tools (10.3%). Other expectations focused on improved communication, access to resources, prevention initiatives, and expanded service features, including animal-related services. Responder expertise was the most frequently expected quality (69.9%), followed by active listening (16.4%) and clear language (12.1%). Additional expectations included responsive and efficient responders, availability of English-speaking staff, and kind, supportive interactions. Clear, adapted recommendations were prioritised by 56.1% of respondents, while 38.0% expected rapid guidance regarding the need for hospital care. French was the preferred language for 84.9% of respondents, and telephone calls were the preferred communication modality (94.1%), although interest in text messaging (24.5%) and video calls (16.7%) was also reported. Some respondents also expressed interest in in-person consultations. Additionally, 81.7% expressed interest in follow-up calls after home monitoring advice. Preferred outreach channels included social media (48.6%), websites (27.5%), and short videos (19.3%) (Table 5). Among unaware respondents, television (44.3%) and social media, particularly Facebook and Instagram (both 38.6%) were more frequently preferred (Table 2). Overall, suggestions focused on improved access to clear and bilingual information, expectations regarding service availability, responder expertise and communication quality, and expanded communication modalities and outreach strategies (Supplementary Table S5).

## Discussion

### Interpretation of findings

The survey identified meaningful gaps in public awareness, expectations, and use of the CAPQ services despite high overall awareness. Unawareness was more common among non-North American respondents, non-French speakers, and those not working in healthcare, suggesting that demographic and cultural factors may influence exposure to poison center services. Regional analyses further showed that both absolute counts and within-region proportions can inform outreach priorities, although these findings should be interpreted cautiously given small subgroup sizes. Among respondents who had experienced a poisoning event, many contacted emergency services rather than CAPQ, including some who were already aware of the center, suggesting incomplete understanding of its role or limited trust in using it as a first resource. Expectations were similar across groups, with nurses and pharmacists most commonly expected to respond rapidly, which aligns with CAPQ operations. Although satisfaction among prior users was high, communication remained an area for improvement, particularly regarding language accessibility, empathy, and clarity during stressful situations. Interest in social media, short-video outreach, and alternative communication tools also suggests that digital strategies could strengthen awareness and accessibility.

### Comparison to previous studies

Our findings align with prior studies showing that poison centers are often underrecognized among certain populations. Several studies have reported underutilization of poison control centers among Black and Hispanic populations in the United States [7–10]. Consistent with these observations, our study found that individuals from ethnic minority backgrounds were significantly less likely to be aware of poison control services, reflecting broader trends of underuse among immigrant and minority groups. Language barriers are another key determinant of poison center use. In the U.S., individuals with limited English proficiency are less likely to access these services [8, 9, 11]. Similarly, in our study, non-native French speakers were more commonly found among those unaware of the CAPQ, highlighting the need for multilingual outreach strategies in Quebec. Unlike research identifying lower poison center use in rural areas [12], we did not observe a clear rural–urban divide, possibly due to variation in exposure or sample representativeness.

Alternative communication tools like text messaging and online chat have shown potential to improve access to poison centers, particularly for those facing barriers to calling [13]. In our study, interest in such tools was noted, especially among respondents unfamiliar with the CAPQ, although their feasibility and cost-effectiveness in Quebec would need further evaluation. Pharmacy-based outreach strategies, such as labels on prescription bags, have been associated with increased awareness and user satisfaction [14] and could represent a practical avenue for the CAPQ. Furthermore, public education campaigns that clearly explain the scope of services, responder qualifications, and confidentiality have also been linked to greater awareness and trust [8, 11], consistent with the gaps in service understanding observed among some respondents in our study. Previous studies of poison and information center services consistently report high overall user satisfaction, with communication quality, accessibility, and clarity of advice identified as key drivers of positive experiences. They also highlight the importance of understandable, user-adapted communication and timely, expert responses as central components of perceived service quality [7, 11, 15]. Consistent with our findings, this suggests that efforts to maintain or improve service quality should primarily focus on strengthening communication practices and accessibility, as these appear to be the most influential determinants of user satisfaction.

### Strengths and limitations

This study provides novel insight into public awareness and expectations of the CAPQ services and is among the first to assess both awareness and user perceptions in the general population. Inclusion of public perspectives beyond clinicians directly involved in poisoning events is a key strength.

Nonetheless, the findings should be interpreted in light of several limitations. The sample size was adequate for descriptive analyses but limited statistical power and the precision of subgroup estimates, which remain exploratory. Voluntary participation may have introduced selection bias, with possible underrepresentation of remote regions and overrepresentation of healthcare workers despite targeted outreach efforts. Nevertheless, because the healthcare worker category was broadly defined, prior CAPQ awareness or use did not necessarily occur within the context of direct patient care. Prior CAPQ use was also similarly distributed between healthcare and non-healthcare workers, suggesting that satisfaction evaluations were not confined to healthcare professionals and thereby limiting potential bias related to professional background. Information bias, including recall and social desirability bias, remains possible, although confidentiality and anonymity were emphasized to mitigate this risk.

### Health system implications

These findings support the importance of poison centers as accessible first-line resources for the management of acute toxic exposures and highlight the need to improve public understanding of when and how to use them. Greater awareness of the CAPQ services may help reduce unnecessary emergency department visits for low-risk exposures and support timely access to expert advice. The results also emphasize that communication quality is central to user experience, particularly during stressful situations in which reassurance, empathy, and clear instructions are essential. Strengthening multilingual communication tools and ensuring that recommendations are adapted to users’ needs may improve both accessibility and trust. For clinicians, these findings reinforce the value of promoting poison center services to patients and families as part of poisoning prevention and safe home management.

### Research implications

This study addresses an important gap by providing provincial data on public awareness, expectations, and satisfaction with the CAPQ, extending beyond prior clinician-focused evaluations. It identifies specific demographic and linguistic disparities in awareness, highlighting populations at risk of underutilization. The study also shows that awareness does not necessarily translate into use, as many aware respondents still turned to emergency services during poisoning events. These findings support the need for future research to better understand the factors shaping help-seeking decisions, including trust, perceived urgency, and communication barriers. Larger and more representative studies will also be important to confirm these patterns and to evaluate whether targeted outreach and different communication strategies improve appropriate poison center use.

## Conclusion

This survey highlights high satisfaction among users of the Centre antipoison du Québec while identifying gaps in public awareness and service expectations. These findings provide insights for strengthening outreach, accessibility, communication, and follow-up practices to better align poison control services with public needs. Future initiatives will include the development of a bilingual public-facing website and a mobile application offering accessible, user-oriented resources for poisoning management. In addition, efforts will focus on expanding the CAPQ’s presence on social media platforms, including Facebook and Instagram.

## Supplementary Information

Below is the link to the electronic supplementary material.

Supplementary file1 (PDF 197 KB)

## Data Availability

The data that support the fi ndings of this study are available by contacting the authors.
