# Supplementary material for: Public awareness and satisfaction with the centre antipoison du quebec (CAPQ): a population-based survey
Source: CJEM. 2026 Jun 28;28(10):929–38. doi: 10.1007/s43678-026-01186-3 (PMC13658303; doi:10.1007/s43678-026-01186-3)
Supplement: Supplementary file 1 [file 43678_2026_1186_MOESM1_ESM.pdf]

Supplementary Table S1. Survey Flow Diagram

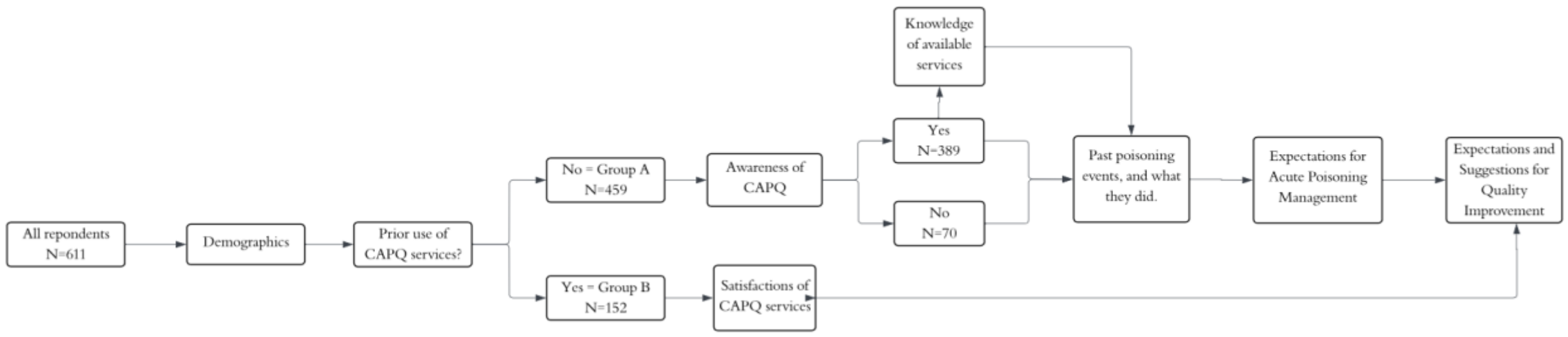

Supplementary Table S2. Regional distribution of awareness of the Centre antipoison du Québec (CAPQ)

Previously Awareness of the CAPQ	Yes (n=541)	No (n=70)	Total (611)
**Area of Residence in Quebec**			
Bas-Saint-Laurent (01)	5 (100%)	0	5
Gaspésie-Îles-de-la-Madeleine (11)	10 (100%)	0	10
Saguenay-Lac-Saint-Jean (02)	14 (100%)	0	14
Nord-du-Québec (10)	4 (100%)	0	4
Capitale-Nationale (03)	220 (86.3%)	35 (13.7%)	255
Chaudière-Appalaches (12)	68 (88.3%)	9 (11.7%)	77
Mauricie (04)	53 (98.1%)	1 (1.9%)	54
Centre-du-Québec (17)	42 (97.7%)	1 (2.3%)	43
Estrie (05)	11 (91.7%)	1 (8.3%)	12
Montréal (06)	39 (81.3%)	9 (18.8%)	48
Laval (13)	9 (75%)	3 (25%)	12
Lanaudière (14)	10 (76.9%)	3 (23.1%)	13
Laurentides (15)	11 (73.3%)	4 (26.7%)	15
Montérégie (16)	20 (87%)	3 (13%)	23
Outaouais (07)	10 (100%)	0	10
Abitibi-Témiscamingue (08)	6 (100%)	0	6
Côte-Nord (09)	9 (90%)	1 (10%)	10

Supplementary Table S3. Prior CAPQ use among healthcare and non–healthcare workers

**Prior use of CAPQ services**		
	Healthcare workers	Non-healthcare workers
Number of participants (n = 152)	77 (50.7%)	75 (49.3%)

Supplementary Table S4. Summary of qualitative user comments regarding Centre antipoison du Québec services

**Tableau résumé des commentaires sur le service offert par le CAPQ**	
**Commentaires sur l'écoute accordée aux appels**	
Positif	Négatif
Excellente écoute	Je suis tombé sur une boîte vocale me demandant d'appeler info santé, alors que c'était info santé qui m'avais dit de vous appeler... assez ordinaire.
Excellent service	La personne a l'appel n'avait aucune courtoisie de plus suivait un protocole dans se soucier de ce que la personne vivait aucune empathie avec et de plus condescendante se disant infirmière et menaçant de raccrocher la ligne prenant les choses personnelles. Un gros ego au bout du fil qui ne faisait pas les bonnes questions. Aucun soucis de la personne au bout du fil.
Les renseignements étaient clairs et facilement applicable. L'accès à votre service a été rapide et efficace.	J'ai été en état de panique et angoisse et la dame qui m'a répondu était indifférente coupe la parole et donne des leçons de moral et je lui ai demandé une question elle a dit je sais pas je me sentais pire après parler à elle.
Efficacité	Utilisé en tant que professionnel donc pas d'attente, maison me rapporte un temps d'attente long parfois.
L'agent était à l'écoute et il savait répondre à nos besoins!	C'est important que ce soit plus rapide d'après moi puisque c'est un service gouvernemental pour lequel on paie et on devrait avoir le droit de pouvoir parler à quelqu'un sans avoir à attendre 1-2h.
Rassurant (3)	Très antipathique. Le service à la clientèle n'est clairement pas sa passion.

Excellente écoute et réponse!	Personne très condescendante
Dans un contexte professionel	On m'a fait sentir comme si ma raison d'appel était ridicule. IL est vrai que c'était une situation mineure mais je préférais m'informer au cas où et j'ai eu l'impression que j'avais appelé pour rien. PErsonne au bour du fil limite bête avec moi.
Vraiment super écoute et sans jugement	
Toujours été bien servie.	
L'agente qui nous a répond a fait un excellent travail.	
Claire et efficace. L'employée a bien compris ce qui me stressant et m'a bien aidée à identifier le produit que j'avais ingéré.	
Calme	
La personne a bien pris le temps de traiter ma situation.	
Super approche et travail. Très apprécié de toute l'équipe des soins!	
Il y a plusieurs années alors que je travaillais à Info-Santé, j'ai joint le centre anti-poison à plusieurs reprises. J'ai toujours eu de bonnes réponses.	
Professionnel, facile à comprendre car formulait de façon normale pour expliquer les termes techniques, rassurant.	
La préposée m'a répondu avec courtoisie et professionnalisme et a sue répondre adéquatement à mon problème.	
On m'a bien renseignée à toutes les fois où j'ai appelé.	
Rapidité de réponse, clarté des informations reçues et rassurance sans jugement	
Infirmière à l'urgence pendant 25 ans, j'ai toujours eu un excellent service.	
Service professionnel	
Excellent service, empathique, rapide et aidant.	
Excellent service	

The call was picked up extremely quickly, for which I am very grateful and satisfied with. I think it is great to get immediate attention and guidance from specialists as to the severity of the situation and what next steps I can take.	
Commentaires sur le **professionnalisme** manifesté par l'intervenant	
Positif	Négatif
Très professionnelle (4)	Aucun professionalisme
Commentaitres rassurants	Pas toujours agréable
Excellent. Nous avons été rassurés.	Condescendante
La personne était très calme ce qui était rassurant.	J'ai été en état de panique et angoisse et la dame qui m'a répondu était indifférente coupe la parole et donne des leçons de moral et je lui ai demandé une question elle a dit je sais pas je me sentais pire après parler à elle.
Super. J'ai aimé aussi les informations données pour mon arrivée à l'hôpital.	They spoke a bit fast which makes it harder to follow sometimes.
Extremely professional and caring.	
C'était vraiment parfait.	
Toujours bien servie.	
S'est assuré que l'on maîtrise la situation avant de mettre fin a l'appel.	
Tout s'est très bien déroulé. Les explications étaient claires et rassurantes.	
Calme	
Toujours très bon service. Jamais de manque de respect ou d'impatience ce qui fait diminuer de beaucoup le stress .	
Rassurant	
L'intervenant était très calme, rassurant	
Je me sentais en confiance.	
Professionnel, facile à comprendre car formulait de façon normale pour expliquer les termes techniques, rassurant.	
Très adéquat	
**Commentaires concernant la clarté des recommandations**	

Positif	Négatif
Réponse très appropriée	Parle incroyablement vite, À peine le temps de noter.
Parfait	Parfois très (trop?) exhaustifs et long délais d'observation pour des intox somme toutes mineures. Des conseils sur "Quoi surveiller" et "quand rappeler" serait peut-être plus bénéfiques dans certains cas que de nommer toutes les possibilités de complication avec les traitemetns associés, alors que le risque est somme toute minime dans la plupart des cas.
Very clear.	Peu être mieux informer et prendre soin de mieux diriger la personne.
Compréhensible	Parfois nécessité à ce qu'on synthétise les recommandations et le plan de suivi.
Très claires, répondent bien aux questions.	
Réponses claires et rassurantes	
Professionnel, facile à comprendre car formulait de façon normale pour expliquer les termes techniques, rassurant.	
Conseils clairs, très détaillés et rassurants	
Excellent	
**Commentaires concernant l'applicabilité des recommandations**	
Positif	Négatif
Recommandation suivie	Pas adapté à la réalité urgence/manque de temps/manque de ressources.
Rassurante	Parfois nous voulons seulement une petite information complémentaire, sans nécessairement faire tout l'historique du patient.
Bon écoute, solution adéquate	
Adéquat	
Applied readily to our situation.	
Ça m'a permis d'éviter d'aller à l'urgence.	
Claire	
On tenait compte du contexte de l'appel . Ex: j'avais besoin d'information sur un antidote	

d’un produit chimique utilisé dans une entreprise.	
Oui les recommandations ont été pertinentes et applicables facilement	
On a bien compris mon besoin et ma situation.	

Supplementary Table S5. Respondent expectations and suggestions for quality improvement of Centre antipoison du Québec services

Avez-vous des suggestions pour améliorer le service
Commentaires quelquefois « paternalistique » de l’infirmier/infirmiere.
Si j'appelle et qu'on ne peut pas prendre mon appel immédiatement, j'aimerais pouvoir laisser mon numéro et qu'on me rappelle plutôt qu'être obligé de rester en ligne à me faire répéter que mon appel est important.
Faire de la publicité : tv, journaux ,radio, et par nos pharmacies sous forme de feuillets explicatifs et même dans les cliniques.. (7)
Le centre devrait avoir une info-lettre pour info sur Ia façon de réagir dans des circonstances précises (e.g.: piqûre de tique).
Tout est dans les délais de réponses qui se doivent d’être rapide….urgence. (4)
C'est important que la personne qui nous réponds soit un professionnel qui connaît bien tous les aspects du centre antipoison.
Avoir accès à une personne qui s'exprime clairement dans ma langue maternelle car je devine que je serai plutôt nerveux lorsque j'appellerai au Centre antipoison du Québec.
Avoir quelqu'un compétant au bout du fil qui est vif d'esprit.
Sitte web accessible avec fiche détaillées sur traitements appropriés autre que appeler le centre.
Lien sécurisé pour accéder a votre site web.
Est-ce qu'une application mobile serait appropriée, ne serait-ce que pour les coordonnées et renseignements de base ?
J'avais besoin d'informations sur le site web et je n'en trouvais pas en anglais. Heureusement j'ai utilisé un traducteur pour m'aider. Si la situation avait été urgente j'aurais téléphoné et je sais que j'aurais eu l'informations.
Mise à part la «vibe» et le débit de parole de l’infirmière, incroyable service. Aucuns délais et informations justes et pertinentes à la situation.
Un suivi de l’appel est une bonne idée.
Coordonnées géographiques inaccessibles. Organisation inhabituelle du site. Recherches plus compliquées, résultats moins nombreux qui au final, obstacle à la curiosité intellectuelle.
Une formation adéquate car le répondant est sur la ligne de front en mesure d'éteindre le feu, par la suite être dans une écoute attentive et faire les bonnes questions et rassurer la personne sur l événement par des conseils adéquats et diriger la personne rapidement au bon endroit. De plus suivre un protocole nom de la personne.

Un service de clavardage ou texto serait très apprécié. (2)
Réseaux sociaux pour prévention et sensibilisation. (2)
L'intervenant doit qd même parler plus poliment et ne reproche pas les gens et surtout être gentille et empathique pour calmer quelqu'un ou est en état de panique .
Numéro de téléphone plus facile à retenir à trois chiffre ex: 777 (4)
Proposer 2 options téléphoniques, soit une pour intoxication URGENTE (temps d'attente moins de 3 minutes) et une semi-urgente, de type le danger n'est pas immédiat, puis un rappel disant que si il ne peut pas y avoir d'attente ou si l'état de santé de la personne se dégrade pendant l'attente de l'appel, contacter les services d'urgences (911).
Appelez 911 pour nous si un danger est détecté .
Proposer une version PDF, Excel ou papier de la page des antidotes : https://www.ciusss-capitalenationale.gouv.qc.ca/antidotes.
Avoir un site web avec des recommandations pour des cas typiques.
Avoir les aimants de frigidaire pour l'accès facile au numéro de téléphone.
Using the user's preferred language. (3) English or French. Available documentation in both languages in printed form. the survey should include comments boxes in several questions to provide more information.
Site web disponible en anglais.
